# LncRNA MDRL Mitigates Atherosclerosis through miR-361/SQSTM1/NLRP3 Signaling

**DOI:** 10.1155/2022/5463505

**Published:** 2022-09-21

**Authors:** Ling You, Yanjie Zheng, Jing Yang, Qian Hou, Lianxia Wang, Yan Zhang, Chunxia Zhao, Ruiqin Xie

**Affiliations:** ^1^Division of Cardiology, The Second Hospital of Hebei Medical University, Shijiazhuang, China; ^2^Institute of Hypertension and Department of Internal Medicine, Tongji Hospital, Tongji Medical College, Huazhong University of Science and Technology, Wuhan, China

## Abstract

**Objective:**

Long non-coding RNAs (lncRNAs) play many important roles in gene regulation and disease pathogenesis. Here, we sought to determine that mitochondrial dynamic related lncRNA (MDRL) modulates NLRP3 inflammasome activation and apoptosis of vascular smooth muscle cells (VSMCs) and protects arteries against atherosclerosis.

**Methods:**

*In vivo* experiments, we applied LDLR knockout (LDLR^−/−^) mice fed the high-fat diet to investigate the effects of MDRL on atherosclerosis. *In vitro* experiments, we applied mouse aortic smooth muscle cells to determine the mechanism of MDRL in abrogating NLRP3 inflammasome and inhibiting cell apoptosis through miR-361/sequentosome 1 (SQSTM1) by TUNEL staining, quantitative RT-PCR, western blot, microribonucleoprotein immunoprecipitation, and luciferase reporter assay.

**Results:**

Downregulated MDRL and increased NLRP3 were observed in mouse atherosclerotic plaques, accompanied with the increase of miR-361. The results showed that MDRL overexpression significantly attenuated the burden of atherosclerotic plaque and facilitated plaque stability through inhibiting NLRP3 inflammasome activation and cell apoptosis, and vice versa. Mechanically, MDRL suppressed NLRP3 inflammasome activation and VSMC apoptosis via suppressing miR-361. Furthermore, miR-361 directly bound to the 3'UTR of SQSTM1 and inhibited its translation, subsequently activating NLRP3 inflammasome. Systematic delivery of miR-361 partly counteracted the beneficial effects of MDRL overexpression on atherosclerotic development in LDLR^−/−^ mice.

**Conclusions:**

In summary, MDRL alleviates NLRP3 inflammasome activation and apoptosis in VSMCs through miR-361/SQSTM1/NLRP3 pathway during atherogenesis. These data indicate that MDRL and inhibition of miR-361 represent potential therapeutic targets in atherosclerosis-related diseases.

## 1. Introduction

Atherosclerosis is a leading cause of cardiovascular and cerebrovascular diseases [1]. It is widely accepted that pro-inflammatory cytokines impair endothelial function and initiate atherosclerosis. Once the initial process is completed, both biochemical and biomechanical stimuli promote plaque development and rupture [2]. The pathological mechanisms of vascular smooth muscle cell (VSMC) dysfunction include aberrant proliferation, migration, apoptosis, and differentiation. Accumulating evidence indicates that excessive apoptosis of VSMCs can drive vascular remodeling and the progression of atherosclerosis [3]. Current study demonstrated that oxidative low-density lipoprotein (ox-LDL) caused a cascade of inflammation and VSMC apoptosis through activation of NLRP3 inflammasome [4], which triggered caspase-1-mediated cleavage of pro-interleukin (IL)-1*β* and pro-IL-18, and secreted mature forms of these mediators from cells [5]. Thus, it is urgent to explore the candidate factors mediating the inhibition of dual signaling pathways and being promising therapeutic strategies for atherosclerosis.

Numerous studies have showed that long non-coding RNAs (lncRNAs) have pivotal functions in a cell-specific manner such as chromatin stabilization, RNA processing, and protein modification [6] Cytosolic lncRNAs could act as competing endogenous RNAs (ceRNAs), which interact with miRNAs and regulate the expression of miRNA target genes. Simion et al. [7] revealed that knockdown of VINAS attenuated atherosclerotic plaque in LDLR^−/−^ mice through inhibition of inflammation in endothelial cells (ECs). Moreover, Ni et al. [8] found that lncRNA CARMN directly interacted with serum response factor (SRF) to regulate the phenotypic switch of VSMCs via RNA pulldown and mass spectrometry analysis, eventually aggravating atherosclerosis. Recently, Wang et al. [9] identified lncRNA MDRL (mitochondrial dynamic related lncRNA) as a key regulator of mitochondrial fission via directly binding to miR-361. In this regard, MDRL overexpression reduced myocardial infarction via suppressing mitochondrial fission and apoptosis in cardiomyocytes [9]. However, major mechanistic gaps remain in the understanding of regulatory lncRNA MDRL involved in vascular injury and atherosclerosis.

In this study, we sought to investigate the role of MDRL in the development of atherosclerosis. We also determined whether MDRL participated in the regulation of NLRP3 inflammasome and apoptosis through sponging miRNAs or proteins in VSMCs. Collectively, these findings provide further insights into lncRNA-mediated VSMC function in atherosclerosis.

## 2. Methods

### 2.1. Mouse Studies

According to the National Institutes of Health guidelines, all mice were maintained and cared for under controlled environment (21 ± 2°C and a 12-h light/dark cycle). All mouse studies performed here were approved by the Institutional Animal Care and Use Committee at the Second Hospital of Hebei Medical University. Eight-week-old male LDLR^−/−^ mice purchased from GemPharmatech Co., Ltd. (Nanjing, China) were fed *ad libitum* with normal diet (ND) or western high-fat diet (HFD) for 12 weeks. Thereafter, mice were sacrificed to harvest the aorta tissues for the following analysis.

### 2.2. Cell Culture and Cell Transfection

HEK-293T cells from the Cell Bank at the Chinese Academy of Sciences (Shanghai, China) were cultured in Dulbecco's modified Eagle's medium (DMEM) high glucose medium (Hyclone Laboratories Inc., UT, USA) supplemented with penicillin (100 units/ml, Hyclone, USA) and fetal bovine serum (FBS, 10%, Hyclone Laboratories Inc., UT, USA), at 37°C and 5% CO_2_ in a humidified chamber.

Mouse aortic smooth muscle cells (MSMCs) were isolated from C57BL/6J mice. Cells were maintained in DMEM containing 10% FBS and 2 mM glutamine and passaged every 3-4 days. MSMCs at Passages 2-4 were used for further study.

As shown in Table S1, silencing RNAs (siRNAs), miRNA mimics or inhibitors, and corresponding controls were designed and synthesized by Genepharma Co., Ltd. (Suzhou, China). Oligonucleotides (50 nM) were transfected into HEK-293T cells or MSMCs using Lipofectamine 3000 (Invitrogen, CA, USA) according to the manufacturer's instructions. Cells were harvested for qRT-PCR or western blot analysis after 48 h of transfection. MDRL cDNA sequences were subcloned, and then gene expression was amplified from the lentiviral vectors. To explore the effect of MDRL on cell function and miR-361, MSMCs and HEK-293T cells were transfected and divided into four groups: scramble siRNA (MDRL-sc), MDRL-siRNA, control adenoviral, or adenoviral MDRL.

### 2.3. RNAscope

The aortas were isolated and harvested from ND or HFD-treated LDLR^−/−^ mice to explore the expression of MDRL in normal arteries and atherosclerotic plaques. RNAscope probes of mouse MDRL and all reagents are purchased from Advanced Cell Diagnostics, Inc. (CA, USA). Paraffin-embedded tissues of mouse aortic roots underwent fixation, dehydration, embedding, cutting, and floating. Sections with a thickness of about 5 *μ*m were deparaffinized, dehydrated, and subjected to antigen retrieval for 15 min at 90°C. After sections were treated with protease III (15 min at 40°C), probes were hybridized for 2 h at 40°C followed by a signal amplification. Subsequently, nuclei were stained with DAPI, and then we performed the signal detection using a RNAscope Multiplex Fluorescent Reagent kit. Images were captured using the Confocal laser scanning microscopy (Leica, SP8, Germany).

### 2.4. Injection of Recombination Adeno-Associated Viruses (rAAVs) and miR-361

Adeno-associated virus serotype 8 vectors for expression of the murine MDRL (rAAV-MDRL), control vectors (rAAV-CTR), scramble vectors (rAAV-scramble), and short hairpin RNA to block MDRL (rAAV-shMDRL) were synthesized by GeneChem Corporation (China). Mice were given intravenous injection of rAAV-CTR or rAAV-MDRL (1x10^12^ viral genome particles) via tail vein and then fed with HFD for 12 weeks. To investigate the effect of knockdown of MDRL on atherosclerosis, LDLR^−/−^ mice were given intravenous injection of rAAV-scramble or rAAV-shMDRL (1x10^12^ viral genome particles) via tail vein and then fed with HFD for 12 weeks [10]. Similarly, miRNA non-specific control (miR-NC) or miR-361 mimics was incubated with Lipofectamine 3000 (Invitrogen, CA, USA) at room temperature for 30 minutes. Mice were injected with miR-NC or miR-361 (5 nmol) weekly for 12 weeks via tail vein as previously described [11].

### 2.5. Analysis of mRNA by Quantitative RT-PCR

Total RNA was extracted from aortic tissues and cells using TRIzol reagent (Invitrogen, USA), and reverse transcribed using PrimeScript RT Master Mix (TaKaRa, Japan). Quantitative RT-PCR (qRT-PCR) was performed with the SYBR Premix Ex TaqTM Kit (Takara, Japan) using a Light Cycler 480 II system (Roche, Switzerland). GAPDH was used as an internal control to normalize gene expression. The sequences of primers are listed in Table S1.

### 2.6. Western Blot

Briefly, aortic tissues and cells from different groups were harvested and lysed with lysis buffer containing the protease inhibitor cocktail. Then, protein was evaluated by SDS-PAGE electrophoresis and transferred to PVDF blotting membrane. The primary antibodies used as follows: NLRP3 (15101, Cell Signaling Technology, USA), SQSTM1 (sequentosome 1, 39749, Cell Signaling Technology, USA), and *β*-tubulin (66362-1-Ig, Proteintech, USA).

### 2.7. Histochemical and Immunohistochemical Staining

Formalin-fixed, paraffin-embedded sections (5 *μ*m thick) of mouse hearts with aortic roots were stained with H&E staining for cap thickness, and Masson's trichrome for collagen as previously described [12]. Images were evaluated by Olympus BX50 microscope and analyzed using the NIH Image J software.

Immunohistochemical staining was performed as previously described. The sections were incubated with primary antibodies against NLRP3 (1 : 200, Abcam, USA) at 4°C overnight, followed by secondary antibody before staining with DAB Kit (ZSGB-BIO, China). Sections incubated with species-matched IgG alone were used as negative controls.

Quantification of atherosclerotic lesions was performed as previously reported [13]. Briefly, the heart sample containing the aortic root was embedded in OCT and frozen at −80°C. Serial 7-*μ*m-thick cryosections from the aortic sinus were mounted on masked slides. Then, the cryosections containing atherosclerotic plaques were quantified with Oil Red O staining following standard protocol as described. Atherosclerosis in the aortic root cryosections was quantified using Image J.

### 2.8. TUNEL Assay

Apoptosis of tissues and cells was measured by TUNEL assay. Apoptotic detection kit (Roche, Switzerland) was used to investigate DNA fragments in the nucleus in situ according to the manufacturer's instructions. Fluorescence microscopy was used to capture images. TUNEL-positive cells and total cells were counted, and the ratio was calculated.

### 2.9. RNA Immunoprecipitation (RIP) Assay

According to the manufacturer's instructions, RIP was performed using a Magna RIP RNA-Binding Protein Immunoprecipitation kit (Millipore, USA) [14]. Briefly, 2 × 10^7^ cell lysates were incubated with RIP immunoprecipitation buffer containing magnetic beads conjugated with anti-Ago2 antibody (ab186733, Abcam, USA) and negative control normal mouse IgG. Then, immunoprecipitated RNAs were isolated and detected using qRT-PCR to determine the enrichment of binding targets, and then the products were pulled down to agarose gel electrophoresis.

### 2.10. Dual-Luciferase Reporter Gene System

To determine the binding among non-coding RNA and mRNA, the target DNA sequence was inserted into pGL3-basic luciferase reporter vector (Promega, USA). HEK-293T cells (1 × 10 [5]) were transfected with miRNA mimics/control and luciferase plasmid together. After 48 hours of transfection, the activity of luciferase reporter was assessed through Dual-Luciferase Reporter Assay (Promega, USA). Relative luciferase activity was normalized to Renilla activity.

### 2.11. Statistical Analysis

The data were analyzed using GraphPad Prism 7.0 software (USA). All continuous variables that complied with normal distribution were presented as the mean ± the standard error of the mean (*SEM*). Experiments were performed using at least three biologically distinct replicates. Student's *t* test (two-tailed) was used for comparisons of two groups, and one-way ANOVA with Bonferroni correction was used for multiple comparisons. Significant differences were determined at *P* < 0.05.

## 3. Results

### 3.1. MDRL Expression Was Decreased in Atherosclerotic Lesion, Accompanied with Upregulated NLRP3 Expression

**Figure 1:**
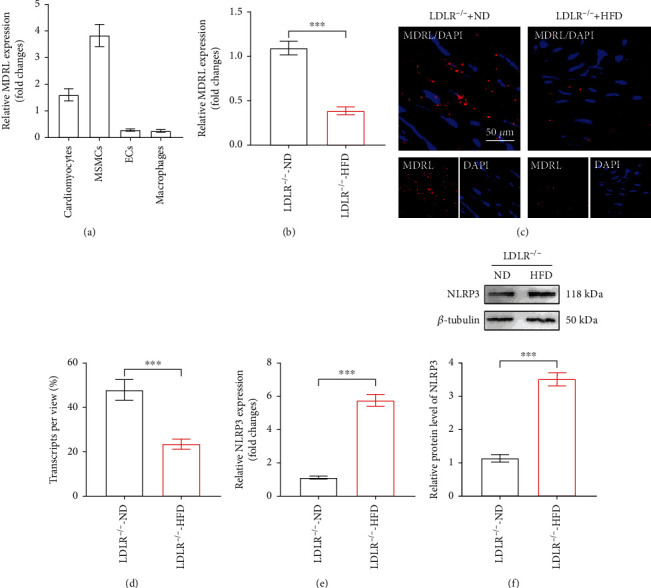
MDRL is abundant in VSMCs and decreased in atherosclerotic lesions. (a) The expression levels of lncRNA MDRL in different cell types were analyzed by qRT-PCR. ^∗∗∗^*P* < 0.001 vs cardiomyocytes group. (b) The expression levels of MDRL in aortas of LDLR^−/−^ mice fed with normal diet (ND) or high-fat diet (HFD) were analyzed by qRT-PCR. (c) Confocal microscopy of MDRL in aortic roots of LDLR^−/−^ mice fed with ND or HFD. Scale bar: 50 *μ*m. (d) Quantitative analysis of MDRL transcripts in aortic roots of LDLR^−/−^ mice fed with ND or HFD. (e) The expression levels of NLRP3 in aortas of LDLR^−/−^ mice fed with ND or HFD were analyzed by qRT-PCR. (f) Representative images and quantification of NLRP3 protein in aortas of LDLR^−/−^ mice fed with ND or HFD. Data are presented as mean ± SEM. ^∗∗∗^*P* < 0.001 vs LDLR^−/−^-ND group.

To investigate the involvement of MDRL in atherosclerosis, we initially examined the expression of MDRL in bone marrow-derived macrophages, MSMCs, and primary pulmonary endothelial cells and found that MDRL was predominantly expressed in MSMCs (Figure 1(a)). Moreover, significant decreased MDRL expression was observed in aortas isolated from HFD-fed LDLR^−/−^ mice than ND-fed LDLR^−/−^ mice (Figure 1(b)). Further, RNAscope results confirmed the reduced MDRL expression in HFD-fed LDLR^−/−^ mice and also demonstrated that MDRL was presented primarily in the cytoplasm (Figures 1(c) and 1(d)). Given the NLRP3 inflammasome-mediated inflammation and pyroptosis in the regulation of atherosclerosis, we also confirmed the changes of NLRP3 during atherogenesis. Consistently, NLRP3 expression was substantially upregulated in aortas of HFD-fed LDLR^−/−^ mice by qRT-PCR and western blot (Figures 1(e) and 1(f)).

### 3.2. MDRL Regulated Atherogenesis in LDLR^−/−^ Mice

**Figure 2:**
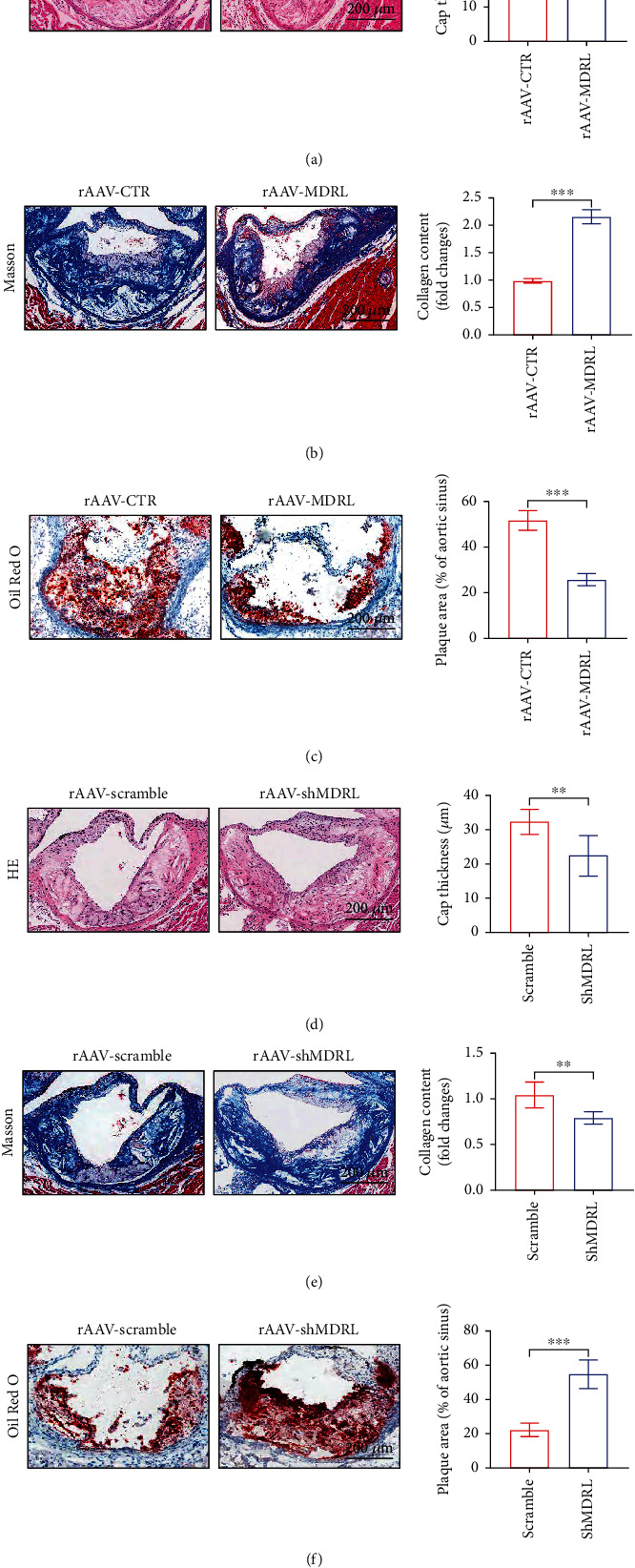
MDRL alleviates atherosclerosis in LDLR^−/−^ mice fed with HFD. (a) Representative images of HE-stained aortic roots from rAAV-CTR- or rAAV-MDRL-treated LDLR^−/−^ mice and quantification of fibrous cap thickness. (b) Representative images of Masson-stained aortic roots from rAAV-CTR- or rAAV-MDRL-treated LDLR^−/−^ mice and quantification of collagen content. (c) Representative images of Oil red O-stained aortic roots from rAAV-CTR- or rAAV-MDRL-treated LDLR^−/−^ mice and quantification of plaque area. (d) Representative images of HE-stained aortic roots from rAAV-scramble- or rAAV-shMDRL-treated LDLR^−/−^ mice and quantification of fibrous cap thickness. (e) Representative images of Masson-stained aortic roots from rAAV-scramble- or rAAV-shMDRL-treated LDLR^−/−^ mice and quantification of collagen content. (f) Representative images of Oil red O-stained aortic roots from rAAV-scamble- or rAAV-MDRL-treated LDLR^−/−^ mice and quantification of plaque area. Scale bar: 200 *μ*m. Data are presented as mean ± SEM. ^∗^*P* < 0.05, ^∗∗^*P* < 0.01, ^∗∗∗^*P* < 0.001 vs rAAV-CTR or rAAV-scramble group.

To determine the effect of MDRL overexpression in pathological context, LDLR^−/−^ mice were treated with rAAV-CTR or rAAV-MDRL. Quantification of atherosclerotic plaques by hematoxylin/eosin staining indicated an increase in cap thickness in the aortic sinus of MDRL-transfected versus CTR-transfected LDLR^−/−^ mice (Figure 2(a)). Masson staining showed increased collagen contents in LDLR^−/−^ mice transfected with rAAV-MDRL compared to those with rAAV-CTR (Figure 2(b)). Furthermore, in comparison with control group, MDRL-transfected LDLR^−/−^ mice developed smaller lesions in aortic sinus (Figure 2(c)). Conversely, inhibition of MDRL by rAAV-shMDRL contributed to an increase in cap thickness and collagen content of the aortic sinus (Figures 2(d) and 2(e)). Knockdown of MDRL by rAAV-shMDRL enhanced the development of atherosclerotic plaques in aortic sinus compared to rAAV-scamble-treated LDLR^−/−^ mice (Figure 2(f)).

**Figure 3:**
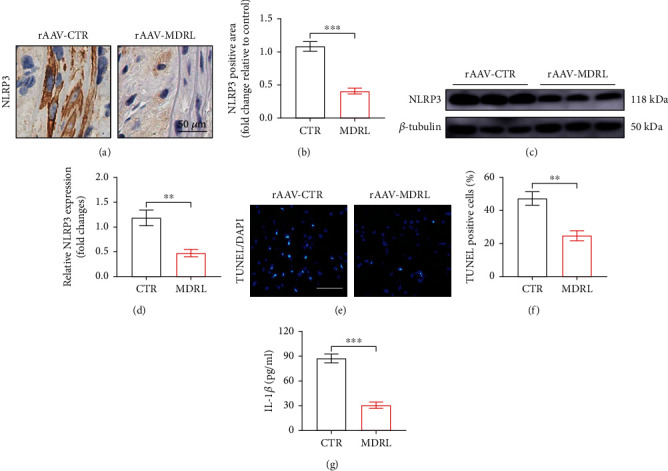
MDRL attenuates NLRP3 inflammasome and apoptosis in plaques of LDLR^−/−^ mice fed with HFD. (a) Representative images of IHC staining for NLRP3 in aortic roots from rAAV-CTR- or rAAV-MDRL-treated LDLR^−/−^ mice. Scale bar: 50 *μ*m. (b) Quantification of NLRP3 positive area in aortic roots. (c and d) Western blots showed the expression levels of NLRP3 protein in aortas from rAAV-CTR- or rAAV-MDRL-treated LDLR^−/−^ mice. (e) Representative images of TUNEL staining (stained with green) in aortic roots from rAAV-CTR- or rAAV-MDRL-treated LDLR^−/−^ mice. Scale bar: 50 *μ*m. (f) Quantification of TUNEL-positive cells in aortic roots. (g). Serum concentration of IL-1*β* from rAAV-CTR- or rAAV-MDRL-treated LDLR^−/−^ mice. Data are presented as mean ± SEM. ^∗∗^*P* < 0.01, ^∗∗∗^*P* < 0.001 vs rAAV-CTR group.

Since the development of atherosclerotic plaques contributed to NLRP3 activation [15], we determined the effects of MDRL on NLRP3 activation. LDLR^−/−^ mice transfected with rAAV-MDRL displayed an approximate 62.41% reduction in the percentage of NLRP3 positive area within aortic plaques relative to control group (Figures 3(a) and 3(b)). In line with this finding, augmentation of MDRL significantly attenuated NLRP3 expression in aorta of LDLR^−/−^ mice (Figures 3(c) and 3(d)). Immunohistochemical analyses of aortic sinus cross sections showed that, compared with control mice, those in MDRL-transfected LDLR^−/−^ mice had reduced TUNEL-positive cells (Figures 3(e) and 3(f)). Correspondingly, serum concentration of IL-1*β* was lower in MDRL-transfected group compared to control group (Figure 3(g)). Taken together, these data suggest a protective role of MDRL against the development of atherosclerotic plaques.

### 3.3. MDRL Inhibited NLRP3 Inflammasome and Apoptosis through miR-361

**Figure 4:**
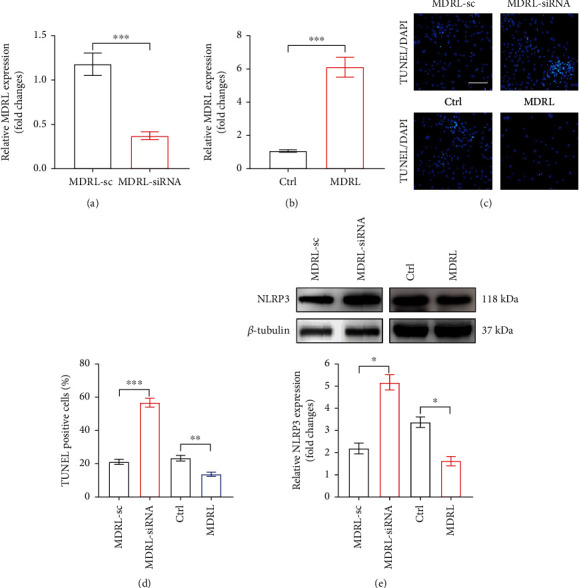
MDRL regulates NLRP3 inflammasome and apoptosis in VSMCs. (a) The expression of MDRL in MSMCs transfected with siRNA (MDRL-siRNA) or scramble (MDRL-sc) were analyzed by qRT-PCR. (b) The expression of MDRL in MSMCs transfected with adenoviral MDRL (MDRL) or control adenovirus (ctrl) was analyzed by qRT-PCR. (c) Representative images of TUNEL staining in MSMCs transfected with MDRL-sc, MDRL-siRNA, ctrl, or MDRL. Scale bar: 50 *μ*m. (d) Quantification of TUNEL-positive cells in MSMCs in the indicated groups. (e) Western blots showed the expression levels of NLRP3 in MSMCs in the indicated groups and quantification. The relative expression of NLRP3 was normalized to housekeeping gene *β*-tubulin in corresponding lane. Data are presented as mean ± SEM. ^∗^*P* < 0.05, ^∗∗^*P* < 0.01, ^∗∗∗^*P* < 0.001 vs MDRL-sc or ctrl group.

To validate the effect of MDRL on NLRP3 inflammasome and apoptosis, we first evaluate the efficiency of MDRL knockdown and overexpression in MSMCs. As expected, the expression of MDRL was significantly downregulated by siRNA (Figure 4(a)), whereas transfection with adenoviral MDRL significantly augmented MDRL expression in MSMCs (Figure 4(b)). While decreased MDRL significantly promoted MSMCs apoptosis, adenoviral MDRL resulted in a reduction of MSMCs apoptosis (Figures 4(c) and 4(d)). Moreover, knockdown of MDRL caused increased expression of NLRP3, and adenoviral MDRL mitigated NLRP3 expression in MSMCs vice versa (Figure 4(e)).

**Figure 5:**
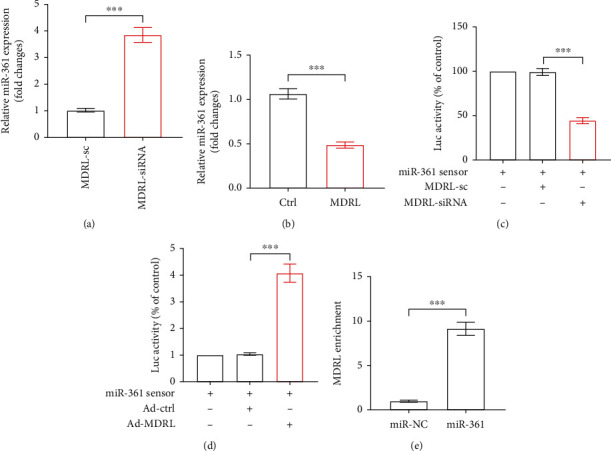
MDRL acts as an endogenous sponge to miR-361. (a) The expression levels of miR-361 in MSMCs transfected with siRNA (MDRL-siRNA) or scramble (MDRL-sc) were analyzed by qRT-PCR. (b) The expression levels of miR-361 in MSMCs transfected with adenoviral MDRL (MDRL) or control adenovirus (ctrl) were analyzed by qRT-PCR. (c) Luciferase activities were measured in HEK-293T cells transfected miR-361 sensor luciferase reporter vector, along with MDRL-siRNA or MDRL-sc. (d) Luciferase activities were measured in HEK-293T cells transfected miR-361 sensor luciferase reporter vector, along with ctrl or MDRL. (e) MDRL binds to miR-361. Biotin-conjugated pulldown assay and qRT-PCR were performed in MSMCs. Data are presented as mean ± SEM. ^∗∗^*P* < 0.001 vs MDRL-sc or ctrl or miR-361 sensor group.

Next, we investigated whether miR-361 acted as an effector of MDRL in VSMCs and NLRP3 inflammasome activation. As shown in Figure 5(a), the expression of miR-361 was significantly increased after transfection of MDRL-siRNA in MSMCs, whereas overexpression of MDRL markedly inhibited miR-361 expression (Figure 5(b)). To explore whether MDRL as a ceRNA competitively bound to miR-361, we transfected the miR-361 sensor luciferase reporter vector, along with MDRL-siRNA or adenoviral MDRL in HEK-293T cells. Compared with scramble siRNA group, we detected a significantly reduced luciferase activity of miR-361 sensor in MDRL-siRNA group (Figure 5(c)). By contrast, a prominent enhancement of luciferase activity was observed in adenoviral MDRL group (Figure 5(d)). We then employed biotin-conjugated pulldown assay in MSMCs, finding that miR-361 could pull down MDRL (Figure 5(e)). These data confirm that MDRL acts as an endogenous sponge lncRNA to interact with miR-361 and blocks miR-361 expression.

### 3.4. MiR-361 Provoked NLRP3 Inflammasomes via Weakening SQSTM1

**Figure 6:**
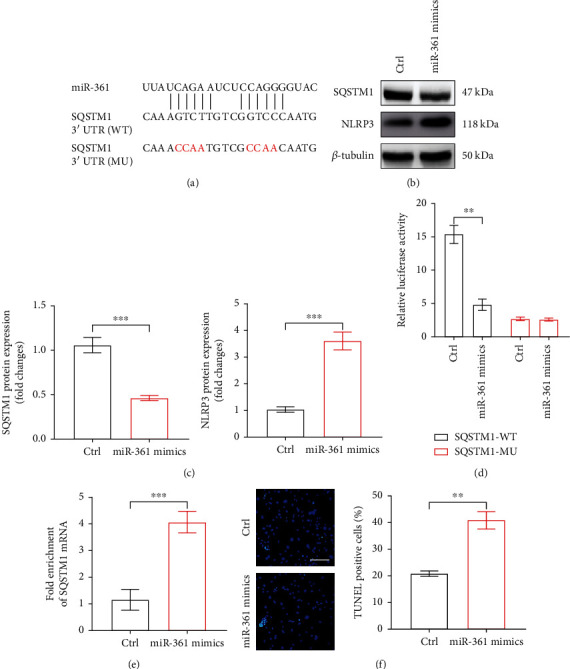
miR-361 binds to SQSTM1 and suppresses SQSTM1/NLRP3. (a) The putative targeting site of SQSTM1 and miR-361 was predicted by Targetscan and PicTar. (b) SQSTM1 and NLRP3 protein expression in MSMCs transfected with miR-361 mimics or control (ctrl). (c) Quantification of SQSTM1 and NLRP3 protein expression on western blots. (d) Luciferase activities were measured in HEK-293T cells transfected with SQSTM1-WT or SQSTM1-MU luciferase reporter vector, along with ctrl or miR-361 mimics. (e) MiR-361 binds to MDRL. Biotin-conjugated pulldown assay and qRT-PCR were performed in MSMCs. (f) Representative images of TUNEL staining in MSMCs transfected with ctrl or miR-361 mimics, and quantification of TUNEL-positive cells. Scale bar: 50 *μ*m. Data are presented as mean ± SEM. ^∗∗^*P* < 0.001 vs ctrl group.

To explore how miR-361 affects NLRP3 inflammasomes in VSMCs, we applied TargetScan and PicTar to the putative targets of miR-361 related to regulation of NLRP3 inflammasomes. The bioinformatic database exhibited the predicted binding site of miR-361 within the 3'UTR of SQSTM1 (Figure 6(a)). As expected, elevated miR-361 resulted in decreased expression of SQSTM1, accompanied with enhanced NLRP3 expression (Figures 6(b) and 6(c)). To confirm whether SQSTM1 was a direct target of miR-361, luciferase reporter plasmid containing the 3'UTR fragment of SQSTM1 was co-transfected with miR-361 mimics into HEK-293T cells. As illustrated in Figure 6(d), the luciferase activity of SQSTM1 3'UTR was remarkably suppressed by miR-361 mimics, whereas the luciferase activity of mutant binding sequences of SQSTM1 3'UTR was not changed. Likewise, microribonucleoprotein immunoprecipitation identified the interaction between miR-361 and SQSTM1 (Figure 6(e)). We also found that overexpression of miR-361 facilitated VSMC apoptosis detected by TUNEL staining (Figure 6(f)).

### 3.5. MiR-361 Is Required for the Inhibitory Effect of MDRL on Atherosclerotic Development in LDLR^−/−^ Mice

**Figure 7:**
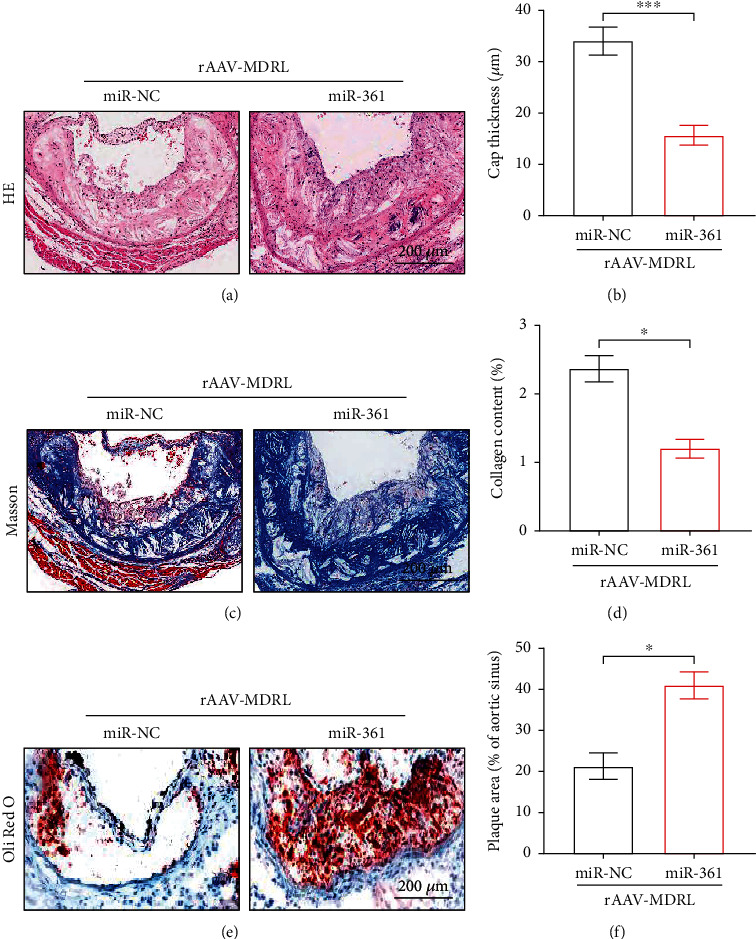
Overexpression of miR-361 counteracts the protective effect of MDRL against atherosclerosis. (a) Representative images of HE-stained aortic roots from control miRNA (miR-NC) or miR-361-treated LDLR^−/−^ mice after intravenous injection of rAAV-MDRL. (b) Quantification of fibrous cap thickness. (c) Representative images of Masson-stained aortic roots from miR-NC or miR-361-treated LDLR^−/−^ mice. (d) Quantification of collagen content. (e) Representative images of Oil red O-stained aortic roots from miR-NC or miR-361-treated LDLR^−/−^ mice. (f) Quantification of plaque area. Scale bar: 200 *μ*m. Data are presented as mean ± SEM. ^∗^*P* < 0.05 vs miR-NC group.

To confirm whether miR-361 was essential for MDRL in regulating atherosclerosis, MDRL-transfected LDLR^−/−^ mice received weekly tail vein injections of miR-NC or miR-361 mimics for 12 weeks. Morphological analyses showed that LDLR^−/−^ mice with both MDRL and miR-361 overexpression had thinner fibrous cap than LDLR^−/−^ mice with only MDRL overexpression (Figures 7(a) and 7(b)). The collagen volume within the lesions was remarkably decreased in miR-361 overexpression mice relative to miR-NC group (Figures 7(c) and 7(d)). Compared to miR-NC group, Oil Red O staining of miR-361-transfected LDLR^−/−^ mice revealed a 2-fold increase in lesion area at the level of the aortic sinus (Figures 7(e) and 7(f)). These data support our hypothesis that MDRL exerts its effect on atherosclerotic development via suppression of miR-361.

## 4. Discussion

Our study reveals that MDRL plays an important role in the development of atherosclerosis. We indicate that MDRL is downregulated in atherosclerotic plaques of LDLR^−/−^ mice. More importantly, overexpression of MDRL alleviates the burdens of plaque and stabilizes plaques. Our *in vitro* mechanistic study shows that VSMC-enriched lncRNA MDRL regulates VSMC apoptosis and inflammation via interplaying with miR-361/SQSTM1 and suppressing NLRP3 inflammasome.

It is accepted that lncRNA confers pleiotropic functions, directly interacting and mediating expression and functionality of DNA, other non-coding RNAs, and proteins. Accumulating studies highlight that lncRNAs participate in atherogenesis and dysfunction of VSMCs [16–18]. For instance, lncRNA CARMN was reported to be abundant in VSMCs and involved in the formation of atherosclerotic plaques, while knockdown of CARMN mitigated VSMC proliferation, migration, and differentiation [8]. In our present study, we identified a novel mechanism through which lncRNA MDRL represses VSMC apoptosis and retards the progression of atherosclerosis. MDRL was initially described to be highly expressed in cardiomyocytes but reduced upon the condition of anoxia followed by reoxygenation [9]. Moreover, MDRL was able to attenuate mitochondrial fission and apoptosis of cardiomyocytes through targeting miR-361 and miR-484 and inhibited the infarct size in mice of ischemia/reperfusion model [9]. Therefore, despite possessing multiple functions, MDRL is likely to confer cardioprotection and atheroprotection. Another important finding of our study was that MDRL alleviated VSMC apoptosis via reducing NLRP3 inflammasomes. As the predominant cells in plaques and tunica media of the vessels, VSMCs are essential for the maintenance of artery structure and function [19]. Apart from phenotypic switching, VSMCs appear to possess universal effects, such as proliferation and apoptosis, which are implicated in vascular remodeling, aortic aneurysm, and atherosclerosis [20, 21]. In this regard, loss and alteration of VSMCs in atherosclerotic plaques exert detrimental effects, causing fibrous cap thinning, necrotic core formation, and plaque rupture [21, 22].

On the other hand, lncRNAs are frequently emerged as ceRNAs for miRNAs. In cardiovascular diseases, Huang et al. [23] reported that the ceRNA network constituted by lncRNA PVT1, miR-3127-5p, and NCKAP1L was involved in the formation of aortic aneurysm. Another study suggested that lncRNA CDKN2B-AS1 as a ceRNA competitively bound to miR-126-5p to upregulate PTPN7, accelerating VSMC apoptosis [24]. Similarly, our findings recognized SQSTM1 as an effector of MDRL/miR-361 ceRNA activity. Previous studies demonstrated that miR-361 functioned as a regulator in glioma aerobic glycolysis and proliferation [25]. In addition, systematic delivery of miR-361 impeded glioma development through suppressing UBR5 [26]. In contrast with these phenomena, our study showed that effective delivery of miR-361 partly offset the protective effect of MDRL on the development of atherosclerosis. These contradictory results are probable to account for the cell-specific potency of miR-361 in cancer cells and VSMCs.

NLRP3 inflammasomes centrally integrate multiple signal inputs in different types of cells and in the pathogenesis of atherosclerosis [27]. In atherogenesis, NLRP3 serves as an important responder to danger signals, including reactive oxygen species, ox-LDL, and cholesterol crystals [28]. NLRP3 inflammasomes drive caspase-1 cleavage, IL-1*β*, and IL-18 maturation and secretion, causing inflammation and pyroptosis [29]. Additionally, activation of NLRP3 inflammasome provoked high mobility group box 1 secretion, promoting VSMC-derived foam cell formation and accelerating atherosclerosis [30]. Current evidence corroborated previous observations supporting a translational strategy for blockage of caspase-1 to prevent VSMC pyroptosis and atherogenesis [31]. There were few known miRNAs targeting assemble and activation of NLRP3 inflammasomes, while hypermethylated miR-145 induced NLRP3 inflammasome through CD137/NFATc1 pathway [32]. Our study demonstrated that MDRL negatively regulates NLRP3 inflammasome via interacting with miR-361 and SQSTM1. Indeed, SQSTM1 served as a bona fide direct target of miR-361 in VSMCs. SQSTM1, nominated p62, is defined as a potent selective autophagy receptor, also engaged in the ubiquitin-proteasome system, cellular metabolism, and apoptosis [33]. Correspondingly, SQSTM1 was reported to interplay with NLRP3 in macrophages, which in turn exerted SQSTM1-dependent autophagic degradation of NLRP3 and inactivation of NLRP3 inflammasomes [34]. Combined with these data, the molecular action of SQSTM1 provides further insight to the relevance of MDRL and miR-361 to activation of NLRP3 inflammasomes and VSMC apoptosis.

## 5. Conclusion

Collectively, the results of our study indicate that the lncRNA MDRL abrogates miR-361 expression, thereby inhibiting VSMC apoptosis via suppression of SQSTM1-dependent NLRP3 inflammasome and consequently retarding atherosclerotic development. These findings establish a new mechanism for controlling gene expression during atherogenesis, providing new insights into lncRNA and miRNA-controlled cellular mechanism.

## Data Availability

The data that support the findings of this study are available upon request from the corresponding authors.

## Supplementary Materials

Supplementary Materials Table S1. Information of primers.
