# Supplementary material for: LncRNA MDRL Mitigates Atherosclerosis through miR-361/SQSTM1/NLRP3 Signaling
Source: Mediators Inflamm. 2022 Sep 21;2022:5463505. doi: 10.1155/2022/5463505 (PMC9519314; doi:10.1155/2022/5463505)
Supplement: Supplementary Materials — Table S1. Information of primers. [file 5463505.f1.docx]

**Table S1. Information of primers.**

| Gene | Primers (5’-3’) |
| --- | --- |
| MDRL | Forward: 5’-CTCCTGCGCTTTAATTTCCAA-3’  Reverse: 5’-TGGCTCTTCCGTAGTTGATGA-3’ |
| GAPDH | Forward: 5’-TGTGTCCGTCGTGGATCTGA-3’  Reverse: 5’-CCTGCTTCACCACCTTCTTGA-3’ |
| miR-361 | Forward: 5′-CCTGGATGT GAAGAAATGT-3′  Reverse: 5′-GTTCTCCGGACCATTCGGCG-3′ |
| U6 | Forward: 5′-GACAGATTCGGTCTGTGGCAC-3′  Reverse: 5′-GATTACCCGTCGGCCATCGATC-3′ |
| SQSTM1 | Forward: 5′-CCTTAATAACTTGGCCGGA-3′ |
|  | Reverse: 5′-CCCG TTAGGATTTCCAG-3′ |
| MDRL-siRNA | 5’-CCTGAGCCCTGAATGCAGA-3’ |
| MDRL pull-down probe | 5’-GCCCAGATGCGCGTGTTCTCTTCCC-3’ |
